# Lateral public health: Advancing systemic resilience to climate change

**DOI:** 10.1016/j.lanepe.2021.100231

**Published:** 2021-10-07

**Authors:** Jan C. Semenza

**Affiliations:** Heidelberg Institute of Global Health, University of Heidelberg, 69120 Heidelberg, Germany

## 1 The COVID-19 health security threat

**Fig. 1:**
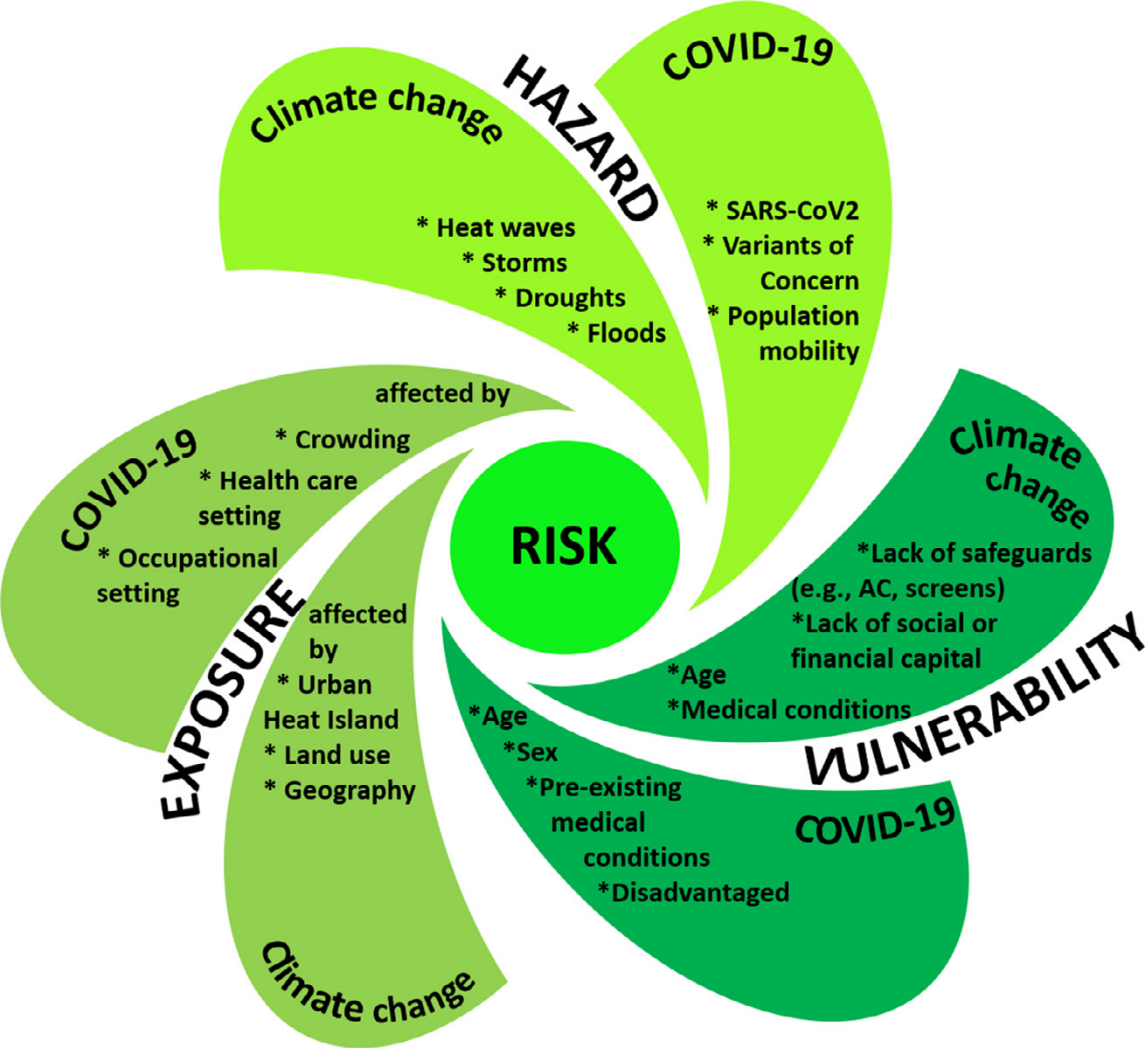
The convergence of two compounding risks: COVID-19 and climate change. Risk is defined as a function of hazard, exposure and vulnerability.

The COVID-19 pandemic, with its staggering mortality and morbidity, has rippled across all facets of life and precipitated the largest synchronized decline in global GDP ever recorded. Countries were caught unprepared for such an acute and expansive health security crisis. This public health emergency has taught us that our health care system cannot cope with multiple crises simultaneously. Compounding risks can exacerbate a challenging health crisis by multiplying the initial impact (Fig. 1). The pandemic has exposed the limits of health care capacity, social inequalities, the vulnerability of marginalized groups, the elderly, and those with pre-existing medical conditions. COVID-19 pummeled even rich countries in Europe, exposing inherent weaknesses in their health care capacity and response to this crisis.

If the response to this acute health security threat was inadequate, how can we then expect European societies to respond to a different, but slow burning, seemingly less urgent, erratic health security threat? Particularly when such an additional, lingering but emerging health threat converges on the COVID-19 crisis (Fig. 1). While initially there was little evidence that COVID-19 would take on such extraordinary proportions, the warnings for the arrival of climate change as an accelerating health security threat have been ample; indeed, climate change has already arrived.

## 2 The climate change health security threat

Climate change is one of the chief public health threats globally [1]. Since the 1960s, we have witnessed a mounting number of climate and weather-related events world-wide, with a 35% increase since the 1990s alone [2]. Every year, climate change-related disasters claim countless deaths, injuries, and livelihoods, such as the July 2021 record rainfall and associated flooding in Western Europe with over 200 fatalities; the global burden of climate-sensitive health outcomes is projected to increase. In Europe, excess deaths are projected to increase during heat waves, particularly among older individuals, due to several factors such as diabetes, obesity and cardiovascular events; a temperature-related range-expansion of certain vector-borne diseases such as Lyme disease already occur and is projected to continue, along with higher incidence rates of certain food and water-borne diseases [3], [4], [5]. The frequency, duration and/or severity of extreme events in Europe are also projected to increase, including storms, heavy rainfall, floods, droughts, wildfires or sea level rise, which can result in a sequence of events that result in a succession of system failures [5]. For example, sea level rise presents risks for settlements in low-elevation coastal zones; flooding poses risks for critical infrastructure, particularly if it affects first responders and hampers their rescue ability; and heavy precipitation can overwhelm water-treatment and distribution systems resulting in large-scale water-borne outbreaks [5]. Thus, extreme weather episodes can precipitate cascading impacts, through a sequence of secondary events in natural and human systems that can result in physical, natural, social or economic disruption due to existing societal vulnerabilities. Even a relatively minor climate hazard can result in a cascade of downstream events when risks are causally connected, with one triggering the next. It can potentially generate an unforeseen sequence of system failures with major public health consequences that need not be in proportion to the initial trigger. For example, a health care facility in a floodplain can easily be inundated by a downpour when it is needed the most, affecting both hospital patients and those seeking care.

Climate change exposes existing vulnerabilities in society and acts as a threat multiplier [6]. Heat waves disproportionally affect the elderly, poor, and marginalized individuals with pre-existing medical conditions, which put them also at risk for COVID-19 (Fig. 1). Mosquito-borne disease outbreaks affect vulnerable communities in close proximity to mosquito breeding grounds, that lack screens on windows or access to repellent. Wildfires tend to rapidly burn through industrial forests, planted for their homogeneous and high yield, at much higher temperature and speed, compared to wildfires in old-growth forests; they can smoke out vulnerable communities in their path.

## 3 Lateral public health

How can European societies tackle these inherent vulnerabilities to climate change? Needless to say, the responsibility for preparedness and response rests with governments. But, will such provisions suffice, under compounding health threats (Fig. 1)? The looming climate crisis requires societal change; it requires retooling public health; it requires advancing lateral public health.

Lateral public health aspires at transcending the siloed confines of traditional public health and to engage governmental agencies and non-governmental actors, including commerce, faith-based organizations, civil society, academia, individuals, and communities [7]. Such a dendritic approach builds inter-agency connections to overcome predefined jurisdictions, and agency connections with communities to enhance their resilience. The core of lateral public health is to develop community capacity for climate change risk reduction through social capital by connecting parties unequal in power and access (e.g. government with community members through linking social capital) [7]. Traditionally, public health operates within the confines of government and vertically administers programs to populations-at-risk. This approach has proven highly successful in tackling single disease issues such as smallpox eradication or polio elimination; however, less so for contextual disease processes with compounding risks, in which social and institutional barriers limit the options for community resilience. In contrast, lateral public health expands beyond sectorial and disciplinary boundaries and alters the dynamics between them, rather than operating with their traditional constituents. It is a transdisciplinary, grassroots approach to public health, grounded in community-based participation in decision-making, preparedness and response and cooperation between sectors. Such an approach is more likely to be effective in the long-run in dealing with multifaceted and global health security threats such as climate change, but also with COVID-19, particularly if they coincide.

Lateral public health aspires to intervene on the three cogwheels of risk: hazard, exposure and vulnerability (Fig. 1). As opposed to vertical public health programs that eliminate one health threat at the time (e.g., polio) but ignore contextual vulnerability (e.g., lack of WASH), this three-pronged scheme is more valuable, because it builds institutional and community resilience to health threats across the three risk domains. Needless to say, such an approach is less immediate, but more effective in the long-run by advancing overall population health and reducing vulnerability to climate change [6,8]. In fact, national implementation of the 17 Sustainable Development Goals requires outreach, organization and coordination between different branches of government and sectors of society. There are a number of successful case studies from across the world that illustrate diverse applications of lateral public health to climate change that are also relevant for Europe (Table 1); two examples are described in more detail below.

**Table 1:** Advancing systemic resilience to climate change through lateral public health approaches; selected examples.

Hazard	Health outcome	Lateral public health approach	Ref
Changes in ecology and weather patterns	Climate-sensitive infectious disease outbreaks	Community-based surveillance for the early detection of an outbreak at community level, rapid detection and response can contain an epidemic. It entails engagement and training of community members in case definitions for climate-sensitive infections such as malaria, acute diarrhea, or cholera and standardized format for reporting (e.g., mobile phones). Community health education regarding disease transmission and treatment modalities can facilitate community participation in outbreak control efforts.	[9,10]
Heat wave	Heat-related mortality and morbidity	Collaboration between communities and institutions to prepare for and respond to heat waves; identification of a lead body to coordinate preparedness and responses; timely forecast of meteorological conditions; community outreach to vulnerable groups to avoid heat exposure.	[11]
Drought and drinking water contamination	Water-borne outbreak	Community-based water harvesting and water purification through low-cost household water chlorination intervention.	[12,13]
Environmental degradation	Civil conflict, physical and mental health	Community and school interventions as well as capacity building initiatives, addressing food and water insecurity collectively through irrigation and stewardship; waste management through community-based clean-up campaigns and solid waste disposal to improve human, animal and environmental health.	[14]
Droughts and food crises	Food insecurity	Engaging the community with government, international organizations, non-governmental organizations and climate scientists to design, develop and implement an early warning system for climatic events (e.g., monsoon, flood, drought). Through monitoring and improved interaction with the community, it can strengthen community resilience to future droughts and food crises from near real-time to long-term.	[15,16]
Pathogens, vectors	Infectious diseases	Through vulnerability, impact and adaptation assessment, information regarding climate sensitive infectious diseases from both health and non-health sectors is collected on: policies and measures; options to manage the health risks; evaluating and prioritizing options; human and financial resource needs; and monitoring and evaluation programs. It can then lead to, for example, upgrading water treatment and distribution systems to avoid waterborne outbreaks or improving urban drainage for vector abatement.	[5,17]

## 4 Practical applications

Flood early warning systems were established in 2008 for two similar communities along the Karnali and Babai rivers in Nepal. In Karnali (in contrast to Babai), a community-based, well-functioning and well accepted formal structure of preparedness and disaster management was established [18]. Community Disaster Management Committees (CDMCs) promoted flood awareness and response capacity to adapt to and mitigate risks. A community-based early warning system was implemented by stakeholders and supported by government and non-governmental organizations. In 2014, a torrential downpour caused the worst floods ever recorded in western Nepal (Fig. 1; hazard). Along the Karnali River, the warning and danger alerts of the early warning system triggered sirens and woke up a sleeping community. They immediately responded as trained, and evacuated people with their larger livestock and movable assets to higher ground (Fig. 1; exposure). They had taken the flood risk seriously and knew how to respond to the alert [19]. Unfortunately, at the Babai river, there was less community buy-in for these preparedness measures, and a number of missteps hampered the response (Fig. 1; vulnerability). The river gauge reader of the early warning system was not able to access the gauge; a delayed alert was triggered when the floodwaters had already engulfed the villages. However, the alert communication chain was not well established and the police did not know whom to transmit the information to. After decisive delays, the chief district officer received the alert but did not appreciate its importance. Eventually, the sirens indicated “warning” as opposed to “danger”, misrepresenting the threat to the community. As a result of this climate disaster, 7 people had died and 767 people displaced from their homes along the Karnali River, compared to 31 and 4,056 respectively, along the Babai River.

Social isolation and lack of social capital (Fig. 1; vulnerability) is a risk factor for heat-related mortality [20]. Enhancing social networks and engaging community members in urban adaptation can both advance social capital and reduce the heat island effect during heat waves (Fig. 1; hazard) [7]. In Portland, OR, a community was engaged in climate change adaptation projects such as collectively building green roofs to reduce rooftop temperatures, establishing urban gardens to cool the air through transpiration, constructing bioswales for catchment of rainwater runoff during storms, starting urban vegetation to increase shading of buildings, and installing cool pavements to reduce heat absorbance of streets. Community members worked alongside city officials, civil engineers and city commissioners to design, permit and implement these features [21]. The process entailed community outreach, informal meetings, design workshops, project development, (building) permitting process, and construction workshops [22]. The interventions were intended to attenuate the negative consequences of climatic events (Fig. 1; exposure to e.g., urban heat, extreme precipitation) by physically improving the built environment. The interventions were subjected to program and process evaluation; the results indicated that such community actions to improve the built urban environment can also enable community capacity in problem solving through improvement in social capital [22,23]. In the context of climate change in urban settings, these findings are important in that they demonstrate that it is possible to simultaneously improve both the physical and social environment, which augments resilience of its residents [23].

## 5 Conclusion

Health permeates all sectors of society and conversely, all other sectors influence health; therefore, health and wellbeing cannot be addressed by one sector alone. The compartmentalization by sectors has served us well in tackling compartmentalized problems. However, specialization commands integration. Complex and contextual problems like climate change with a long-term horizon that lie outside the box of individual sectors do not lend themselves well to be addressed with a sectorial approach. Currently, individual actors may want to collaborate across sectors but there is no institutional incentive to do so. Moreover, there is no process and no format to come to the table. The inadequacy of this disciplinary divide has become particularly apparent during the acute COVID-19 threat; the lack of compliance with non-pharmaceutical measures such as face masks, mobility restrictions or physical distancing indicates a public disengagement with this health threat.

Learning from the COVID-19 response, lateral public health stipulates for a three-tiered approach towards climate change, directed at hazard, exposure, and vulnerability [24] (Fig. 1); it stipulates for a dendritic approach to health security by mainstreaming health aspects into other sectors; and it stipulates for a forum to coordinate different stakeholders, to set the goals and targets and how to achieve them. Such an integrated approach allows for scaling up effective strategies to tackle hazard, exposure and societal vulnerabilities, to offset synergistic amplification of compounding risks (Fig. 1; e.g., by COVID-19). Moreover, lateral public health seeks to avert cascading impacts from a sequence of secondary events that are causally connected, with one event triggering the next. It intends to break these causal chains through transparency and multi-sectorial interventions and through investments in health systems, poverty and inequality reduction. However, most importantly, lateral public health strives for community engagement in decision-making, preparedness and response. The COVID-19 health security threat offers the opportunity to overcome the predicaments of traditional public health by leapfrogging to lateral public health.

## Declaration of interests

Dr. Semenza has nothing to disclose.
